# Time trends in socioeconomic differences in incidence rates of cancers of gastro-intestinal tract in Finland

**DOI:** 10.1186/1471-230X-6-41

**Published:** 2006-12-04

**Authors:** Elisabete Weiderpass, Eero Pukkala

**Affiliations:** 1 Department of Etiological Research, The Cancer Registry of Norway, N-0310 Oslo, Norway; 2 Department of Medical Epidemiology and Biostatistics, Karolinska Institutet, 171 77 Stockholm, Sweden; 3 Institute for Statistical and Epidemiological Cancer Research, Finnish Cancer Registry, Liisankatu 24, FI-00170 Helsinki, Finland

## Abstract

**Background:**

The magnitude of socioeconomic differences in health varies between societies, and over time within a given society. We studied the association between social class and incidence of cancers of the gastro-intestinal tract over time in a large cohort in Finland.

**Methods:**

We studied social class variation among 45–69 year-old Finns during 1971–95 in incidence of cancers of the gastro-intestinal tract by means of a computerized record linkage of the Finnish Cancer Registry and the 1970 Population Census, which included social class data.

**Results:**

There were 2.3 million individuals in the cohort under follow-up, with 1622 cases of cancer of the esophagus, 8069 stomach (non-cardia), 1116 cardia, 408 small intestine, 6361 colon, 5274 rectum, 1616 liver, 1756 gallbladder, and 5084 pancreas during 1971–1995. Cancers of the esophagus, stomach, cardia, gallbladder and pancreas were most common among persons belonging to a low social class. Cancers of the small intestine in males only, colon in both genders, and rectum in females were most common in the higher social classes. Incidence of stomach cancer decreased and incidence of colon cancer increased over time in both genders in all social classes, and the large differences between social classes remained unchanged over time. Incidence rates of cardia cancer did not change substantially over time.

**Conclusion:**

There is a large variation in incidence of cancer of the gastrointestinal tract by social class in Finland. Although much of the observed social class differences probably could be explained by known etiological factors such as diet, physical exercise, alcohol consumption, smoking and exogenous hormone use, part of the variation is apparently attributable to largely unknown factors.

## Background

The magnitude of socioeconomic differences in health varies between societies, and over time within a given society. Previous studies identified associations between low social class and stomach, esophagus and pancreas cancers (both for incidence and mortality). Positive socio-economic gradients (i.e. low risk in low socioeconomic strata) were observed for colon cancer for males and females, both for incidence and mortality [1-3]. No consistent trends according to socio-economic strata were observed in most studies for rectum [1,2], liver [4-6], and gallbladder cancer [4,5]. For esophageal cancer, excess incidence and mortality concentrates in lower socioeconomic classes in many countries in different continents [7-14]; with exception of the US [4,15,16]. The association between lower socioeconomic status and esophageal cancer is observed both for squamous cell carcinomas [17,18] and for adenocarcinomas [19]. For stomach cancer (non-cardia) studies have been highly consistent indicating an increased incidence and mortality among lower socioeconomic status. For cancers of the gastric cardia the association with socioeconomic status is still unclear [20]. Pancreas cancer incidence is not consistently associated with socioeconomic status, although there is a tendency towards higher age-adjusted pancreatic cancer risk in industrialized countries [21,22]. Data for small intestine [23] and biliary tract cancers [24] is very scarce, and the few published studies indicate no clear association with socioeconomic status

Differences in cancer risk between social classes may be interpreted as implications of inequity in the society [25,26]. Furthermore – because lower social classes tend to adopt life habits of higher classe, as happened for example with smoking habits over time – the incidence levels of higher social classes today seem to predict the average level in the whole population in the future [27].

Finland is an industrialized country populated by Caucasians, whose cancer incidence rate patterns and trends probably can be generalized for similar regions. We present here results from a large study carried out in Finland on the association over time between social class and incidence of cancers of the gastro-intestinal tract.

## Methods

### The cohort

The cohort comprised participants born 1906–1945 who participated it the official Population Census organized by Statistics Finland on the last day of 1970 [28]. All residents in Finland were expected to complete a comprehensive questionnaire including demographic and socio-economical questions, among others. The response rate was 98% [29].

A social class classification with four ordinary classes was chosen. This classification results from sociological studies in Finland [30], and was formed on the basis of education, occupation, industrial status and industry groupings [28]. Financially dependent persons (e.g., housewives and students) were classified by the occupation of their supporter.

The four social classes were defined as follows:

I. Managers and other higher administrative or clerical employees, farmers owning more than 50 hectares of land,

II. Lower administrative or clerical employees, smallscale entrepreneurs, farmers owning 15 to 49.9 hectares of land,

III. Skilled and specialized workers, farmers owning 5 to 14.9 hectares of land, and

IV. Labourers, farm and forestry workers, institutions' inmates, farmers owning less than 5 hectares of land, pensioners whose former occupation is unknown.

Persons with unknown social class (1.5% of the total population or 1.0% of the economically active population; mainly farmers and fishermen) were included in social class III, because their behaviours in terms of general mortality and morbidity resembles that of social class III (for details see Pukkala, 1995 [27]).

### Follow-up

The follow-up started on January 1, 1971, and finished at emigration, death or on December 31, 1995, whichever occurred first. Person-years for each social class and sex were counted by five-year birth cohorts (1906–1910,, 1941–1945), and the follow-up period was divided into five-year groups (1971–1975, ..., 1991–1995). The Finnish Cancer Registry has collected data on cancer case incidence in Finland since 1953. Institutions with hospital beds, medical practitioners, and pathological laboratories notify the Registry about cancer cases that are identified. Moreover, Statistics Finland reports to the Register whenever cancer is mentioned on the death certificate. If there are only laboratory notifications or death certificate information from a cancer case, the Registry requests clinical data from the treatment hospital(s).

Linkages between the census, emigration, mortality, and Cancer Registry files were performed using personal identifiers. These unique eleven-digit codes, given to each resident in Finland since 1967, are used in all main personal registers in the country. The magnitude of mistakes due to incorrect personal identification numbers is less than 0.01%, and has a negligible effect on cancer risk estimates [31]. Among eligible cancer cases, 2.3% had to be excluded because the patients were not found in the 1970 Population Census.

### Risk estimates

Cancer risk estimates were calculated for strata defined by sex, social class, calendar period, and birth cohort. We restricted the analyses to birth cohorts aged 45 to 64 years at the beginning of each 5-year observation period because these are the age categories within the economically active Finnish population during 1970 census which could be followed throughout the 25-year observation period of our study. Persons included in the first period (197175) were born in 190625 and in the last period (199195) in 192645. Whenever age is mentioned below, it means the age at the beginning of each 5-year period, although the persons at the end of each five-year follow-up period were actually five years older. For each stratum, two types of indicators of cancer frequency were calculated:

1. Incidence rate: observed number of cases divided by the stratumspecific number of personyears, given per 100,000 person-years. In the figures and text, age-adjusted totals are weighted averages of the four age-group specific incidence rates. The weights were based on the age distribution of all person-years in the study series.

2. Standardized incidence ratio (SIR): ratio of the observed and expected number of cases. The expected number of cases was achieved by multiplying the stratumspecific number of personyears by period and birth cohort specific incidence rate of the total economically active Finnish population of the same sex. Confidence intervals (CI) were defined assuming that the observed number of cases followed a Poisson distribution.

We will present results for primary invasive cancers of the esophagus, stomach, cardia, small intestine, colon, rectum, liver, gallbladder and pancreas.

This study was made with the permission of Statistics Finland, who has the right to give out anonymous linked data for ethically sound research projects.

## Results

There were 2 342 053 individuals in the cohort (1 074 383 men and 1 267 670 women) under follow-up. Of the women 7% were in social class I, 32% in social class II, 47% in social class III, and 14% in social class IV. The respective percentages for the men were 9%, 24%, 54%, and 13%.

### Esophageal cancer

There were 981 esophageal cancers among men and 641 among women. The overall incidence rate was 8.9/10^5 ^among men and 4.7/10^5 ^among women. For esophageal squamous cell carcioma the incidence was 6.23/10^5 ^among men (690 cases) and 3.76 among women (517 cases), while for esophageal adenocarcioma the incidence was 1.05/10^5 ^among men (116 cases) and 0.17 among women (23 cases).

**Figure 1:**
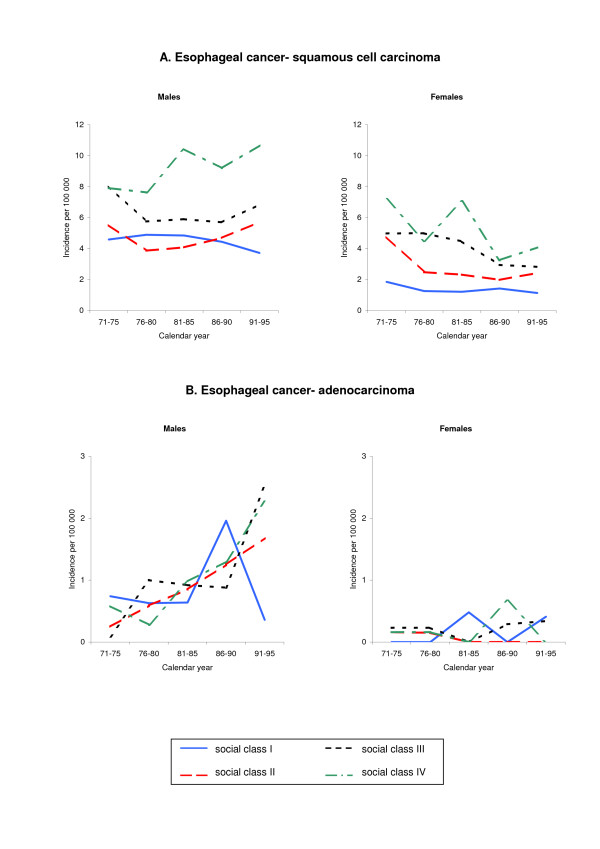
Age adjusted incidence of Esophageal cancers histological types among Finns aged 45–64 at the beginning of each 5-years period between 1971–1995.

Overall, the incidence increased towards lower socioeconomic status (SES) among men from SIR 0.68 in social class I to1.40 in social class IV and among women from SIR 0.44 for social class I to 1.34 in social class IV (Table 1). The social class gradient was very clear for esophageal squamous cell carcinomas: among men the relative difference between the SIRs of social classes I and IV remained similar over time; among women the relative differences between social classes somewhat diminished over time because of the decreasing incidence in social classes II to IV. The incidence of squamous cell carcinoma did not vary substantially by birth cohort among men. Among women, the birth cohorts born before 1920 had esophageal squamous cell carcinoma incidence rates twice as high as the later birth cohorts (Fig. 1A).

For esophageal adenocarcinomas the differences in incidence between social classes were not statistically significant (Table 1); estimates were unstable for women due to small number (Fig. 1B). There was no clear evidence of a birth cohort effect in males or females.

### Stomach cancer – non-cardia

Men had 4861 cases of stomach cancer (non cardia), and women 3208 during the study period.

**Figure 2:**
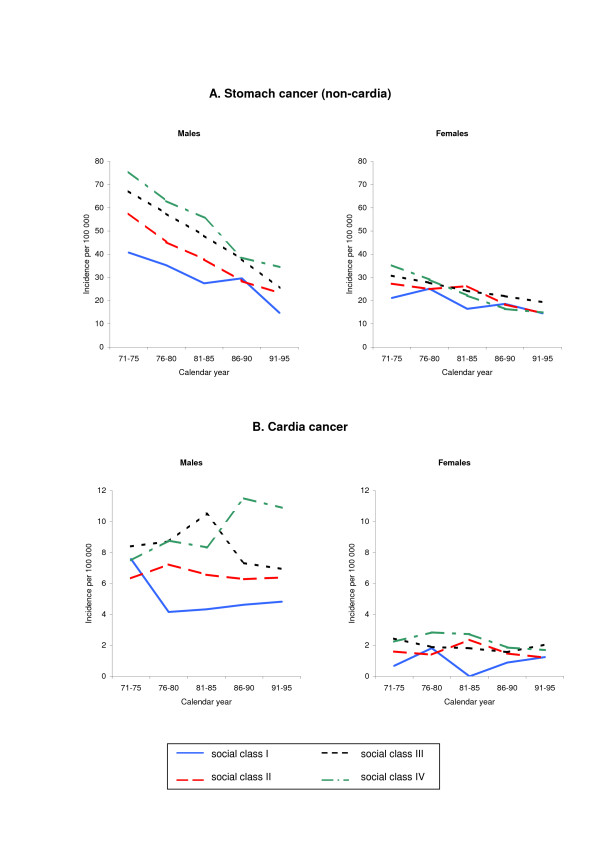
Age adjusted incidence of Stomach non-cardia and Cardia cancers among Finns aged 45–64 at the beginning of each 5-years period between 1971–1995.

The overall incidence rate was 32.5/10^5 ^among men and 16.0/10^5 ^among women. There was a two-fold difference in SIRs between social classes I and IV among males and a much smaller difference among females (Table 2). The relative differences among men remained stable over the calendar period despite the marked decline in incidence rates in all social classes (Fig. 2A). Among females, the decline was strongest in social class IV.

### Cardia cancer

There were 863 cardia cancer cases among men and 253 among women during the follow-up period. The overall incidence rate was 7.8/10^5 ^among men and 1.8/10^5 ^among women. In both genders, there was a gradient with the highest rates in social class IV (Table 2). The incidence over time within each social class was rather stable (Fig. 2B).

### Colon cancer

In the study series 3062 men and 3269 women were diagnosed with colon cancer.

The overall incidence rate was 27.6/10^5 ^among men and 24.0/10^5^among women. The SIRs increased towards higher social classes for both men and women, the difference between social classes I and IV being clearly more marked among men than among women (Table 2).

**Figure 3:**
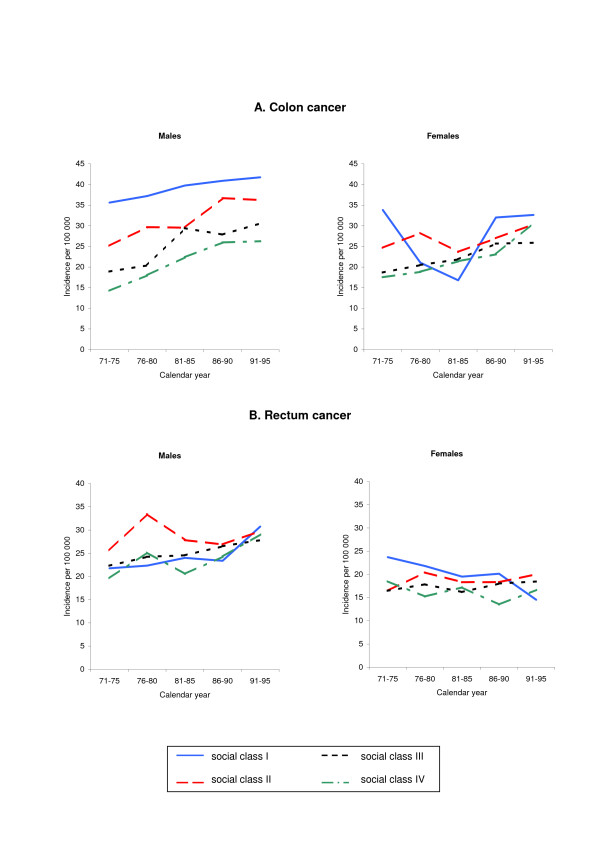
Age adjusted incidence of Colon and Rectum cancers among Finns aged 45–64 at the beginning of each 5-years period between 1971–1995.

The differences between social classes slightly decreased over time among men due to a steeper increase in social class IV than in the other social classes (Fig. 3A). Among women, incidence rates increased in social classes II to IV, while in social class I there was an inconsistent trend (Fig. 3A).

### Rectal cancer

A total of 2855 cases of rectal cancer were reported among men and 2419 among women during the study period. The overall incidence was 25.8/10^5^among men and 17.7/10^5 ^among women.

The incidence was rather similar in all social classes (Table 2) and stable throughout the whole 25 year observation period in all social classes and both genders (Fig. 3B).

### Liver cancer

There were 1029 primary liver cancers diagnosed during the follow-up among men and 587 among women.

**Figure 4:**
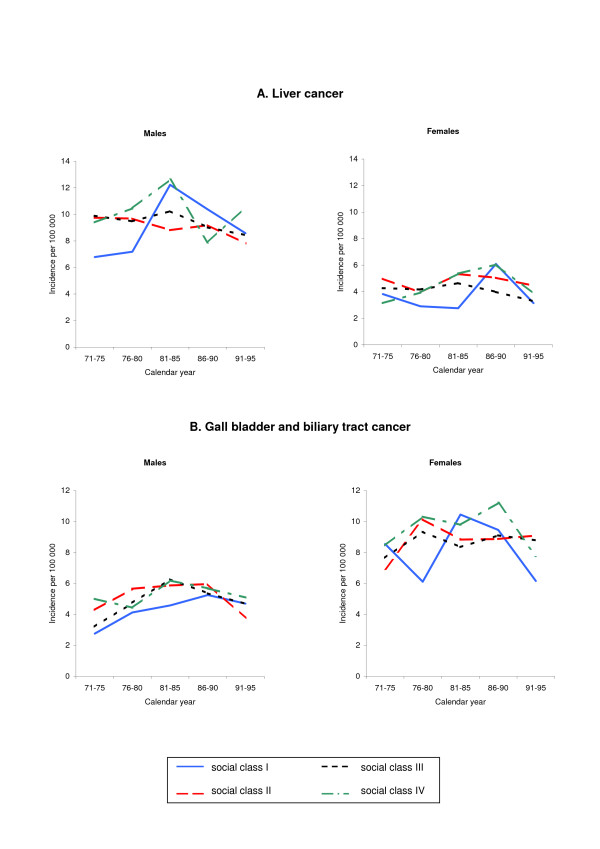
Age adjusted incidence of Liver and Gall bladder and biliary tract cancers among Finns aged 45–64 at the beginning of each 5-years period between 1971–1995.

The incidence rates among men (9.3/10^5^) were higher than among women (4.3/10^5^). There were no variations in SIRs between social classes (Table 3) and no clear increasing nor decreasing trend during the follow-up period in any social class or gender (Fig. 4A).

### Gallbladder and biliary tract cancer

There were 1756 cases diagnosed during the follow-up period (549 among men and 1207 among women). The overall incidence rates were clearly higher among women (8.8/10^5^) than among men (4.9/10^5^). The overall social class trend in both genders indicated slightly lower SIR in social class I than in social class IV (Table 3). In both genders and all social classes incidence rates turned from increase to decrease during our follow-up period (Fig. 4B).

### Small intestine cancer

**Figure 5:**
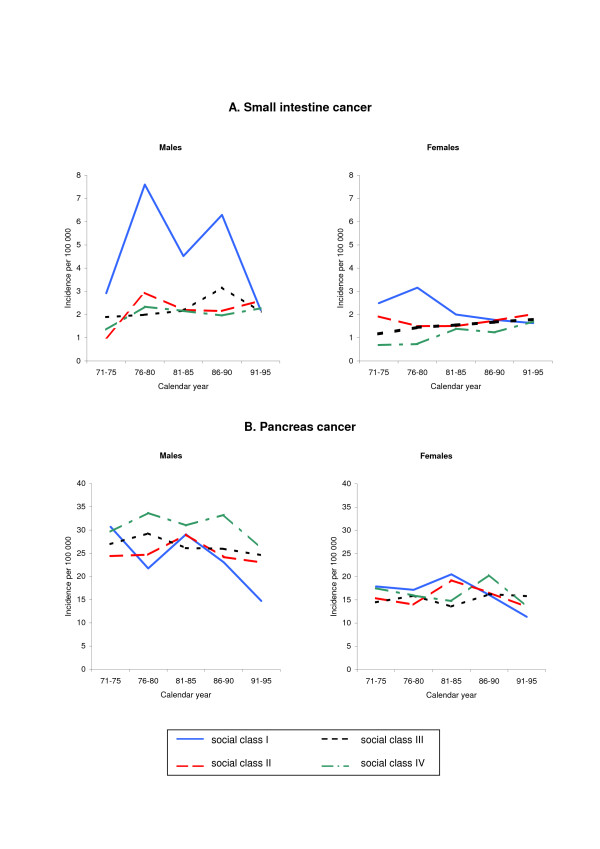
Age adjusted incidence of Small intestine and Pancreas cancers among Finns aged 45–64 at the beginning of each 5-years period between 1971–1995.

A total of 480 cases of small intestine cancer were diagnosed during the study period, 271 in men and 209 in women. The overall incidence rate was 2.4/10^5 ^among men and 1.5/10^5 ^among women. There was no clear evidence of increasing incidence rates over time (Fig. 5A). The SIRs were markedly higher in social class I than in social class IV, especially in males (Table 3). These differences were more evident in the beginning of the follow-up period, and almost disappeared by the end of the observational period (Fig 5A).

### Pancreas cancer

There were in total 2936 pancreas cancers detected among men and 2148 among women during the observation period. The overall incidence rate was 26.4/10^5 ^among men and 15.6/10^5 ^among women.

Among men there was a mild social class gradient with social class I having the lowest SIR while among women there was no difference between social classes (Table 3). In both genders incidence rates were stable over time in social classes II to IV, but in social class I there was a decrease in incidence rates after the mid 1980s (Fig. 5B).

## Discussion

### Overall summary of the results

#### Incidence trends

The incidence rates in the population were increasing for colon cancer and for esophageal adenocarcinomas (among men and women), while decreasing for esophageal squamous cell carcinoma (more evident among women), stomach (non-cardia; both men and women), and in more recent years for gallbladder (men and women) and pancreas (in men and women).

No clear changes in incidence rates were observed for esophageal squamous cell carcinoma (males), cardia (men and women), small intestine (males and females), rectum (men and women), and liver (men and women) cancer.

#### Overall SIR variation by social class

Esophageal squamous cell carcinoma and cancers of the stomach, cardia, and pancreas were more common among persons belonging to lower social classes. For esophageal adenocarcinomas, cancers of the gallbladder, liver and rectum there was no clear social class differences in incidence. Higher social classes had increased incidences of small intestine cancer (specially among men) and colon cancer (in both genders).

#### Changes in social class patterns in cancer incidence over time

There were no changes over time on the social class patterns for esophageal squamous cell carcinomas or adenocarcinomas, and cancers of the stomach, cardia, rectum, liver, gallbladder, and pancreas. The most marked patterns of cancer incidence by social class were observed for stomach cancer (non-cardia) with clearly decreasing incidence trends in all social classes and for both genders. For small intestine cancer the differences between social classes seem to decreased over time, although numbers are small to reach a firm conclusion. For colon cancer, especially among men, there was a large difference sustained between social classes.

### Comparison with previous studies and potential explanations for observed trends

#### Esophagus cancer

For esophageal squamous cell carcinoma we found a higher incidence in the low social classes, and the differences between social classes were rather stable over time. There was a decreasing overall incidence in females and a stable incidence in males in social classes II, III, and IV, and a decreasing incidence in social class I since the mid 1980s. The higher incidence of esophageal squamous cell carcinomas in lower social classes has been reported before in several studies; indeed, there seems to be an inverse gradient in risk, regardless if the population is at high or low risk [32-37]. This social class difference may be explained by well-established risk factors, namely smoking and alcohol consumption [33] and low fruit and vegetable intake [38]. We believe that the decreasing trends of esophageal squamous cell carcinoma in social class I in men could be at least partially explained by trends in smoking (decreasing over time), and diet (increase in fruit and vegetable consumption) among the wealthy in Finland. For women, smoking prevalence is increasing over time in Finland, an observation that is in contrast with our findings of a decrease in esophageal squamous cell carcinoma incidence. Alcohol consumption is not systematically associated with social class in Finland [39]. As in men, consumption of fruits and vegetables has been increasing in Finland, which may have contributed to a decline in incidence rates among women. In Finland, consumption of fruits and vegetables is highest among high social classes. A large number of both case-control and cohort studies has been conducted on the impact of alcohol and smoking on risk of esophagus cancer, mainly esophageal squamous cell carcinoma. The overall results of these studies are quite consistent indicating that both – smoking and alcohol consumption – are independent risk factors for esophageal squamous cell carcinoma, and act synergistically in the causation of this cancer form. The association between tobacco consumption and esophageal squamous cell carcinoma risk seems to be dose dependent, as well as dependent on the duration of the habit [40,41]. The risk of esophageal squamous cell carcinoma drops follow cessation of smoking, and only a small to moderate residual excess risk lasts after than more years after cessation [40,42-44]. There is a linear dose-risk trend of esophageal squamous cell carcinoma with alcohol consumption [35,41,45,46]; the dose of alcohol consumed per day or week appears to be the most important determinant of risk, and not the duration of the habit [41,43,47,48]. In at least one study, restriction of alcohol consumption amongst moderate to heavy drinkers apparently reduced their risk of squamous cell carcinoma of the esophagus [49]. Tobacco and alcohol consumption are strong determinants of risk in both genders [43,50], but the risk estimates are systematically lower among women than among men [43]. Given the lower prevalence of smoking and drinking among women it has been estimated that the proportion of cases that could be attributable to these factors was 90% in men and 29% in women [43]. For fruits and vegetables, there is limited evidence suggesting protection against esophageal cancer; both for squamous cell carcinoma and adenocarcinomas [38].

For esophageal adenocarcinomas incidence rates are overall increasing in both genders, while there was no clear evidence of social class differences over time. The increase in incidence over time is in agreement with studies from the United States [51], Europe [52], Australia [53], and New Zealand [54]. Smoking and alcohol consumption are less strongly associated with risk of esophageal adenocarcinomas as compared with esophageal squamous cell carcinoma. Relative increase in weigh – and not only overweight and obesity – is clearly and strongly associated with the risk of esophageal adenocarcinomas [55,56], while not associated with esophageal squamous cell carcinoma. The secular trend in weight increase among the Finnish population may therefore explain at least in part the increasing trends in esophageal adenocarcinoma. Gastroesophageal reflux disease is the strongest risk factor for esophageal adenocarcinoma [57]; although reflux disease is common amongst obese individuals, it can occur in individuals of all body sizes, and its effect seems to be independent of obesity [57]. Besides some evidence of protection for the consumption of fruits and vegetables [58], no other dietary or nutritional patterns have yet been consistently associated with risk of esophageal adenocarcinomas [33].

#### Stomach cancer

For stomach cancer we found a higher incidence in the lower social classes, and social class differences were rather stable over time. Overall incidence rates decreased rapidly during the study period in both genders. Similar overall declines in overlapping calendar periods were previously reported in several European countries [59] and in the US [60,60,61].

Smoking is a well established risk factor for stomach cancer [62], and there is some indication that the risk of stomach cancer associated with smoking is higher amongst individuals with low intake of fruits and vegetables [63]. Other risk factors for gastric cancer include infection with *Helicobacter Pylori (H. Pylori)*, a diet poor in fruits and vegetables and rich in red meat, in particular processed meat [64,65], pickled food and highly salty food [66]. Infection with *H. Pylori *seem to have an interactive effect with consumption of red meat [64] and possibly with highly salty foods.

#### Cardia cancer

Cancer of the gastric cardia was more common among males and in low social classes. No marked changes overtime was observed in the different social classes, or in the overall incidence rates in the population. An increase in incidence of gastric cardia cancer has been noted in some Western countries [51,67-69] in the same period, coinciding with the increasing trends in esophageal adenocarcinoma [67,70]. In Sweden, however, after a careful classification of all tumors, no increasing trend could be confirmed for cardia cancer [71]. In any case, the incidence curves for gastric cardia cancers are clearly different than those from distal gastric cancer. Again, we could not find similar incidence trend data over time by social class from other countries to compare our data on cardia cancer with. In an independent analysis of the Finnish data [72] described the same overall and gender specific patters as we did. Consumption of read meat seems not to be associated with cardia cancer risk, at least in Europe [64]; while consumption of fruits and vegetables possibly decreases risk. The number of studies on cardia cancer risk factors is relatively small, and the evidence is not firmly established. Improving of life conditions in Finland after the II World War, with consequent decreased endemic infections with *H. Pylori*, together with decrease in prevalence of smoking in the Finnish male population and increasing in consumption of fruits and vegetables may partially explain the decrease incidence of stomach cancer.

#### Colon cancer

For colon cancer we found higher incidences in high social classes, and a slight decrease in social class differences over time. The overall incidence however was clearly increasing in both genders in Finland during the studied period, as it was in most Western countries [73] and countries which are increasingly adopting western lifestyles such as Singapore [74], China (urban areas) [75] and, at very high rates, Japan [76]. In the US incidence rates for colorectal cancer seem to have reached a peak in the early 1980's and started declining afterwards [77]. The nationwide screenings for colorectal cancer in Finland started to be implemented in 2004 by the Finnish Cancer Society [78]. Thus, persons in our analysis have not been screened regularly. The most important established risk factors for colon cancer include increased body weight and obesity and lack of physical activity [79]; both factors increasing markedly in the Finnish population in the last decades and probably explaining at least 1/3 of the secular trends in increasing incidence [79]. Overweight and obesity are more common among low social class persons in Finland; physical activity used to be highest in blue-collar occupations, but it has decreased in the last decades with automation. Leisure-time physical activity is most popular among white-collar professions. Alcohol consumption [80], and intake of red meat, in particular processed meat [81] increase colon cancer risk. Consumption of animal fat, possibly associated with increase incidence of colon cancer, linked to the consumption of meat and fat used to be highest among the richest in Finland, but nowadays the low-fat products are most popular in the highest social classes [82,83]. Intake of fruit and vegetables [38], dietary fiber [84,85], milk and dietary calcium [86] are probably preventive for colon cancer. The proportion of women using hormone replacement therapy, described in several observational studies as decreasing risk for colon cancer, was higher among wealthier women in Finland up to 1980s [87], and the social class differences in use of these drugs disappeared in the 1990s (Dr. Eero Pukkala, personal communication). Part of the irregularity in the social class variation in the incidence rates of colon cancer among women may thus be due to differences in use of exogenous hormones. The social class variation among women was smaller than among men, which fits with the pattern of HRT use among wealthier women in the country.

#### Rectal cancer

For rectal cancer, besides the higher incidence among males than females, we found no clear variations by social class or time period. Similarly, most previous studies found no consistent trend according to social class in men or women [4].

Body size and physical activity are not clearly related to risk of rectal cancer, but diet may have a role as a component in its etiology, although no association has been firmly established so far [79].

#### Liver cancer

For primary liver cancer we found no clear evidence of differences in incidence rates by social class. Previous studies suggested either a negative social class gradient (i.e. increased risk in lower social classes) or no gradient at all [4]. The most common form of liver cancer is hepatocellular carcinoma, for which the major etiological factors have been identified: a viral etiology is conclusively established with chronic infections with hepatitis B and C viruses increasing risk greatly. It has been estimated that about 75% of all hepatocellular carcinomas are caused by infections with hepatitis B or C [24]. Other established risk factors include excessive alcohol consumption, tobacco smoke, and dietary aflatoxin. Finland is a low risk country with very low prevalence of hepatitis B [88] and hepatitis C [89,90] infection [91], and unlike the US [89,92] there is no clear evidence for an increasing prevalence of hepatitis C infections.

#### Gallbladder cancer

In our study, gallbladder cancer was more common among women, and low social classes had higher incidence rates than social class I, with evidence of declining incidence rates in all social classes since the early 1990s. We could not identify other studies with which our data on social class trends could be compared. In a recent analysis of data from several countries around the world there has been a marked global increase in mortality from intrahepatic, but not extra-hepatic, biliary tract malignancies [93]. Little is know about risk factors for gallbladder cancer. The most consistent observation is the history of gallstones or gallbladder disease [24,94-96] and the risk of gallbladder cancer. Fat intake and obesity – although not firmly established as risk factors for gallbladder cancer – are important risk factors for the formation of gallstones. Intake of fruits and vegetables may be protective for gallbladder cancer, but the evidence is sparse [94,97].

#### Small intestine cancer

For small intestine cancer we observed that incidence was higher in privileged social classes particularly in the beginning of the period studied. In an Italian study, no significant differences in social class and risk of small intestine cancer was observed [98]. We observed no substantial changes in incidence rates over time. Our observation of a small decrease in incidence in social class I has only been reported in earlier Finnish data sets [27]. Males had higher rates of small bowel cancer compared to females in the majority of countries, and in the US the incidence seems to be rising among African-American men [99]. A recent study from the England, Wales and Scotland indicate a small increase in incidence rates over the period 1975–2002, and a slightly increased incidence in males as compared to females; no differences in incidence or survival between social classes was reported [23].

Knowledge about the risk factors of small intestine cancer, which is a rare disease, is very limited. Intake of alcohol has been suggested as risk factor in some [100-102] but not all [98,103] previous studies. Other suggested risk factors for small intestine cancer include smoking [98,101,102] and other dietary factors such as intake of heterocyclic amines (from fried foods), sugar, carbonated drinks, salted, cured or smoked foods, bread/pasta/rice, and red meat [98,102,103]. However, for none of these dietary factors the evidence is firmly established. Intake of fruits and vegetables has been described as possibly protective [98], although again, the evidence is scanty.

#### Pancreas cancer

For pancreas cancer higher incidence rates were observed in low social classes among men, although the differences were not very marked, and seemed to be rather stable over time. There was a slight decrease in incidence in social class I in both genders. Elsewhere, pancreas cancer has not been consistently associated with any social class in particular, once the effect of smoking, which is probably the sole known well established modifiable risk factor, is accounted for [4,104,105]. Smoking has been more prevalent among men in lower social classes in Finland for many decades [106]. Smoking was first a habit of highly educated urban women, but since the 1970s it increased among women of the lowest social classes. The social class pattern observed for pancreas cancer – higher risk in the lower social classes disappearing over time – fits with the smoking habits of Finnish women, and is in parallel with the lung cancer epidemic in the country [107].

#### Methodological considerations

The completeness of the Finnish Cancer Registry is over 99% for all malignant solid tumors diagnosed in Finland [108], and accuracy is high. False positive diagnoses are not registered as cancers [109]. The coverage and accuracy of the death and emigration files in Finland is very high. Because of the high coverage and accuracy of the linked files, the cancer risk estimates by social class can be considered reliable, and the large numbers of cases downplay the role of chance even in the case of relatively rare cancer forms.

The census of 1970 was the first computerized census in Finland and was therefore used for this study. Social class was assessed before the cancer diagnoses, i.e., the bias caused by a downward drift in the social hierarchy as a result of disease [110] should have been avoided. Because occupational stability in Finland has been high, the cross-sectional information from 1970 should reflect the social class circumstances from several decades. Social class can be defined in many alternative ways. However, different determinants of social class tend to give parallel results in patterns of general mortality [111] or cancer incidence rates [112-114]. The classification of social class in this study is based on occupational titles. The accuracy of the occupational codes (on which the social class coding is largely based) in Finnish censuses is high: the net error in the occupational codes in the 1980 census was less than 2% [115]. The occupational stability between censuses of 1975 and 1980 and between censuses of 1980 and 1985 was 85–86% in both sexes [115], being highest (96–97%) among high social class workers. Low stability was characteristic for blue-collar workers. However, even if they change work, they do not change their social class.

Most countries lack linkable population-based registries on cancer incidence. Previous studies on social class variation in cancer risk are mostly based on mortality, instead of incidence. This may cause bias, because the principles in definition of the underlying cause of death vary by time, region, and perhaps even by social class or occupation. Mortality from competing causes of death, and survival of cancer patients also varies by social class [116,117]: a positive social class gradient in the incidence rates can change to a negative one in mortality, due to better survival in upper social classes, as demonstrated with the Finnish data for rectal cancer [27].

## Conclusion

Although much of the observed social class differences probably could be explained by known etiological factors such as diet, physical exercise, alcohol consumption, smoking and exogenous hormone use, part of the variation is apparently attributable to largely unknown factors.

Evidently social class variation has to be taken into account as a possible confounder in studies on risk factors for cancers of the gastrointestinal tract. This study shows that there is substantial variation in incidence of cancer of the gastrointestinal tract by social class in Finland. Future studies need to concentrate on the explanation of this variability in incidence.

## Abbreviations

CI confidence intervals

HRT hormone replacement therapy

SES socio economic status

SIR standardized incidence ratio

## Competing interests

The author(s) declare that they have no competing interests.

## Authors' contributions

The article was conceptualized together by Drs. Elisabete Weiderpass and Eero Pukkala.

EP provided the materials and performed the statistical analysis of the data, helped with the interpretation of results, and commented the manuscript.

EW interpreted the data, prepared the tables and figures, wrote the manuscript, and revised it according to the referees comments.

## Pre-publication history

The pre-publication history for this paper can be accessed here:



## Figures and Tables

**Table 1:** Standardized Incidence Ratio of esophagus cancer and histological types of esophageal carcinoma

Cancer site	Esophagus					
	
	All histologies*		Squamous cell carcinoma		Adenocarcinoma	
	
	Obs	SIR 95% CI	Obs	SIR 95% CI	Obs	SIR 95% CI
MEN						
Social class I	61	0.68 0.52–0.88	44	0.70 0.51–0.94	9	0.78 0.36–1.48
Social class II	180	0.79 0.68–0.91	123	0.77 0.64–0.91	25	0.90 0.58–1.33
Social class III	541	1.04 0.95–1.13	378	1.03 0.93–1.14	66	1.07 0.83–1.36
Social class IV	199	1.40 1.21–1.60	145	1.45 1.23–1.70	16	1.05 0.60–1.71
WOMEN						
Social class I	19	0.44 0.26–0.69	13	0.37 0.20–0.64	2	1.20 0.14–4.32
Social class II	117	0.70 0.58–0.84	100	0.74 0.61–0.90	2	0.32 0.04–1.14
Social class III	344	1.11 0.99–1.23	270	1.08 0.95–1.21	14	1.28 0.70–2.14
Social class IV	161	1.34 1.14–1.55	134	1.38 1.16–1.63	5	1.24 0.40–2.89

Esophagus, Esophagus Squamous cell carcinoma and Esophagus Adenocarcinoma among Finnish men and women aged 45–64 at beginning of each five-year follow-up period, and standardized incidence ratios (SIR) with 95% confidence intervals (CI) in 1971–95, by social class and period. Reference: economically active Finnish population.

* The addition of squamous cell and adenocarcinoma cases does not sum up with "total" due to cases with other histological diagnosis.

**Table 2:** Standardized Incidence Ratio of stomach non-cardia, cardia, colon and rectum cancer

Cancer site	Stomach cancer (non-cardia)		Cardia cancer		Colon cancer		Rectum cancer	
	
	Obs	SIR 95% CI	Obs	SIR 95% CI	Obs	SIR 95% CI	Obs	SIR 95% CI
MEN								
Social class I	280	0.67 0.59–0.75	54	0.69 0.52–0.90	394	1.37 1.24–1.51	256	0.98 0.86–1.10
Social class II	957	0.86 0.81–0.92	169	0.84 0.72–0.98	823	1.14 1.07–1.22	736	1.10 1.03–1.19
Social class III	2740	1.06 1.02–1.10	491	1.07 0.98–1.17	1513	0.93 0.88–0.98	1488	0.98 0.93–1.03
Social class IV	884	1.20 1.12–1.27	149	1.19 1.01–1.39	332	0.78 0.69–0.86	375	0.92 0.83–1.02
WOMEN								
Social class I	184	0.82 0.71–0.94	9	0.51 0.23–0.97	273	1.13 1.00–1.27	192	1.10 0.95–1.26
Social class II	804	0.94 0.88–1.01	59	0.88 0.67–1.14	1007	1.11 1.04–1.18	699	1.06 0.98–1.14
Social class III	1620	1.05 1.00–1.10	130	1.06 0.89–1.25	1467	0.94 0.89–0.99	1138	0.98 0.93–1.04
Social class IV	600	1.03 0.95–1.11	55	1.19 0.90–1.55	522	0.92 0.85–1.01	390	0.92 0.83–1.01

Stomach non-cardia, cardia, colon and rectum cancer cases among Finnish men and women aged 45–64 at beginning of each five-year follow-up period, and standardized incidence ratios (SIR) with 95% confidence intervals (CI) in 1971–95, by social class and period. Reference: economically active Finnish population.

**Table 3:** Standardized Incidence Ratio of liver, gall bladder & biliary tract, small intestine and pancreas cancer

Cancer site	Liver cancer		Gall bladder & biliary tract cancer		Small intestine cancer		Pancreas cancer	
	
	Obs	SIR 95% CI	Obs	SIR 95% CI	Obs	SIR 95% CI	Obs	SIR 95% CI
MEN								
Social class I	89	0.97 0.78–1.19	44	0.88 0.64–1.18	47	1.85 0.36–2.46	231	0.87 0.76–0.99
Social class II	229	0.96 0.84–1.09	132	1.03 0.86–1.21	57	0.90 0.68–1.16	647	0.95 0.88–1.02
Social class III	549	1.00 0.92–1.09	287	0.98 0.87–1.10	135	0.94 0.79–1.10	1564	1.00 0.95–1.05
Social class IV	162	1.07 0.91–1.24	86	1.09 0.87–1.35	32	0.84 0.58–1.19	494	1.16 1.06–1.26
WOMEN								
Social class I	37	0.88 0.62–1.22	78	0.91 0.72–1.13	21	1.34 0.83–2.05	158	1.04 0.88–1.21
Social class II	176	1.11 0.951.28	325	1.00 0.89–1.11	65	1.11 0.86–1.42	583	1.01 0.93–1.09
Social class III	267	0.95 0.84–1.07	568	0.98 0.90–1.06	98	0.99 0.80–1.20	1004	0.97 0.91–1.03
Social class IV	107	1.02 0.84–1.23	236	1.09 0.96–1.23	25	0.70 0.46–1.04	403	1.04 0.95–1.15

Liver, gall bladder & biliary tract, small intestine and pancreas cancer cases among Finnish men and women aged 45–64 at beginning of each five-year follow-up period, and standardized incidence ratios (SIR) with 95% confidence intervals (CI) in 1971–95, by social class and period. Reference: economically active Finnish population.
